# Familial Hypercholesterolemia and Lipoprotein(a): A Gordian Knot in Cardiovascular Prevention

**DOI:** 10.3390/metabo12111065

**Published:** 2022-11-04

**Authors:** Amalia Despoina Koutsogianni, Petros Spyridonas Adamidis, Fotios Barkas, Evangelos Liberopoulos, Ta-Chen Su, Shizuya Yamashita, George Liamis, Manfredi Rizzo

**Affiliations:** 1 Department of Internal Medicine, Faculty of Medicine, School of Health Sciences, University of Ioannina, 45110 Ioannina, Greece; 2 Department of Hygiene and Epidemiology, Faculty of Medicine, School of Health Sciences, University of Ioannina, 45110 Ioannina, Greece; 3 1st Propaedeutic Department of Medicine, School of Medicine, National and Kapodistrian University of Athens, Laiko General Hospital, 10679 Athens, Greece; 4 Departments of Environmental and Occupational Medicine and Internal Medicine, National Taiwan University College of Medicine, National Taiwan University Hospital, Taipei 10617, Taiwan; 5 Department of Cardiology, Rinku General Medical Center, Rinku Ourai Kita 2-23, Osaka 598-8577, Japan; 6 Department of Internal Medicine and Medical Specialties, School of Medicine, University of Palermo, 90133 Palermo, Italy

**Keywords:** familial hypercholesterolemia, lipoprotein(a), cardiovascular disease, hypolipidemic treatment, cholesterol

## Abstract

Familial hypercholesterolemia (FH) is the most frequent genetic disorder resulting in increased low-density lipoprotein cholesterol (LDL-C) levels from childhood, leading to premature atherosclerotic cardiovascular disease (ASCVD) if left untreated. FH diagnosis is based on clinical criteria and/or genetic testing and its prevalence is estimated as being up to 1:300,000–400,000 for the homozygous and ~1:200–300 for the heterozygous form. Apart from its late diagnosis, FH is also undertreated, despite the available lipid-lowering therapies. In addition, elevated lipoprotein(a) (Lp(a)) (>50 mg/dL; 120 nmol/L), mostly genetically determined, has been identified as an important cardiovascular risk factor with prevalence rate of ~20% in the general population. Novel Lp(a)-lowering therapies have been recently developed and their cardiovascular efficacy is currently investigated. Although a considerable proportion of FH patients is also diagnosed with high Lp(a) levels, there is a debate whether these two entities are associated. Nevertheless, Lp(a), particularly among patients with FH, has been established as a significant cardiovascular risk factor. In this narrative review, we present up-to-date evidence on the pathophysiology, diagnosis, and treatment of both FH and elevated Lp(a) with a special focus on their association and joint effect on ASCVD risk.

## 1. Introduction

Familial hypercholesterolemia (FH) is caused by mutations in the genes involved in low-density lipoprotein (LDL) catabolism, and is related to premature atherosclerotic cardiovascular disease (ASCVD) [1]. Although available lipid-lowering therapies are effective in reducing LDL-C levels and cardiovascular risk in FH patients, the majority of these are diagnosed late or remain undertreated [2]. On the other hand, elevated lipoprotein(a) (Lp(a); hyperLp(a)), controlled mostly genetically by the *LPA* gene locus, is also associated with increased ASCVD risk [3,4]. Novel therapies targeting apolipoprotein(a), such as antisense oligonucleotide or silent RNAs, are effective in lowering Lp(a), but their cardiovascular benefit is yet to be determined [5]. A considerable proportion of patients with FH is diagnosed with high Lp(a) levels, but there is debate about whether these two entities are associated. A few studies have demonstrated a higher prevalence of hyperLp(a) in genetically or clinically diagnosed FH patients, but others suggest that a considerable proportion of these might have been misdiagnosed due to high Lp(a) levels [6,7,8]. Nevertheless, the co-existence of FH and hyperLp(a) seems to multiply the risk of ASCVD [6].

Herein, we narratively review the available data regarding the pathophysiology, diagnosis, and treatment of both FH and hyperLp(a). Furthermore, we focus on their relationship and joint effect on ASCVD risk.

## 2. Materials and Methods

Relevant studies were identified by searching the MEDLINE, Embase, and CENTRAL databases up to 6 September 2022 using the following terms: familial hypercholesterolemia, lipoprotein(a), cardiovascular disease, hypolipidemic treatment, and cholesterol. The reference lists from these articles were also scrutinized.

## 3. Results

### 3.1. Familial Hypercholesterolemia

#### 3.1.1. Definition and Prevalence of FH

FH prevalence varies depending on the definition used and population studied. Homozygous patients (HoFH) are rare, with an estimated worldwide prevalence of 1:300,000–400,000 [9], while heterozygous FH (HeFH) prevalence is estimated to be ~1:200–300 [10].

#### 3.1.2. Genetics of FH

FH is mostly caused by functional mutations in the following genes: LDL receptor (LDLR), proprotein convertase subtilisin kexin 9 (PCSK9), apolipoprotein B (apoB), and LDLR adaptor protein 1 (LDLRAP1) [11]. Mutations in these genes may impair the LDLR-mediated catabolism of LDL by markedly reducing hepatic LDL clearance and leading to LDL-C accumulation [11]. Among FH patients, 85–90% exhibit mutations in LDLR, 2–4% in PCSK9, 1–12% in apoB, and only a few in LDLRAP1 genes [11,12,13,14].

Considering that FH is an autosomal dominant disorder inherited with a gene-dosing effect, homozygotes are more adversely affected than heterozygotes [9]. It should also be noted that most HoFH patients are compound heterozygotes [9]. This is attributed to a large number of distinct LDLR gene mutations [9]. Therefore, an adult with HoFH is more likely to have inherited genetic mutations in two different LDLRs [9]. True homozygosity more often occurs in cases of consanguineous union between two heterozygotes [9]. Of note, a high prevalence of a limited number of LDLR mutations may occur in a region due to the founder gene effect [9].

#### 3.1.3. Clinical Presentation of FH

If left untreated, males and females with HeFH typically develop coronary heart disease (CHD) before the age of 55 and 60, respectively, while HoFH individuals develop CHD very early in life, and many will die before the age of 20 [15,16].

The characteristic lipid profile of FH patients consists of elevated total cholesterol and LDL-C (usually ≥190 mg/dL for HeFH or ≥500 mg/dL for HoFH) with normal high-density lipoprotein cholesterol (HDL-C) and triglyceride (TG) levels [15,16].

#### 3.1.4. Diagnosis of FH

The diagnosis of HeFH is based on genetic testing or clinical criteria [17,18]. The most commonly used clinical criteria for HeFH are the Dutch Lipid Clinic Network (DLCN) which include patient family history for increased LDL-C levels, personal clinical history of premature ASCVD, the presence of tendon xanthomas, the presence of corneal arcus in a person < 45 years, patient LDL-C levels, and genetic testing, if available [13]. HoFH diagnosis is based on either genetic testing or an untreated/treated LDL-C > 500/300 mg/dL together with the presence of xanthomas before the age of 10 years or untreated LDL-C levels in both parents compatible with HeFH [13].

In the case of FH diagnosis, a screening cascade in all first-degree relatives is strongly recommended, including children beginning from the age of 2 years [10]. It is worth mentioning that, despite its well-documented high prevalence, <5% of those with FH have been diagnosed worldwide and the majority of those are diagnosed over 40 years old [2].

#### 3.1.5. Prognosis and Treatment of FH

At any level of untreated LDL-C, the prognosis for HeFH patients is worse than individuals without FH [19]

Possible ASCVD predictors in FH patients include family ASCVD history, age, gender, smoking, hypertension, type 2 diabetes, hyperLp(a), as well as other potential risk modifiers [1]. These include genetic parameters beyond the traditional FH-causing mutations, parameters of HDL composition and function, inflammation, telomere length in somatic cells, oxidative stress, and hemostasis [1].

Concerning FH treatment, the European Society of Cardiology/European Atherosclerosis Society (ESC/EAS) 2019 guidelines recommend an LDL-C target < 70 mg/dL (1.8 mmol/L) for FH patients without cardiovascular risk factors, and <55 mg/dL (1.4 mmol/L) for those with ASCVD or additional risk factors [10].

Maximally tolerated statin therapy ± ezetimibe is the cornerstone of treatment for FH patients [20]. Other therapeutic agents include PCSK9 inhibitors, bempedoic acid, and bile acid sequestrants [20,21]. In the case of HoFH, lomitapide, evinacumab, and lipoprotein apheresis are additional treatment options [20].

### 3.2. Lipoprotein(a)

#### 3.2.1. Molecular Properties of Lp(a)

Lp(a) consists of an LDL-like particle, in which apoB-100 is linked by a single disulfide bridge to a unique plasminogen-like glycoprotein, known as apolipoprotein(a) (apo(a)) [3,4]. The LDL-like core constitutes a combination of triacylglycerols, phospholipids, and esterified/unesterified cholesterol surrounded by one molecule of apoB-100 [3,4]. Considering its formation, Lp(a) contributes to atherosclerosis, but also exerts inflammatory, oxidative, thrombotic, and antifibrinolytic properties [3,22,23].

#### 3.2.2. Genetics of Lp(a)

About 90% of the Lp(a) level is autosomal dominantly inherited and strongly determined by a single gene, the *LPA* gene [23]. Most individuals express two distinct Lp(a) isoforms [23]. The *LPA* gene, located in chromosome 6q23, is evolutionarily derived from the plasminogen (PLG) gene and remains highly homologous to it [23,24,25,26]. The *LPA* gene, and thus apo(a), consists of two kringle domains: kringle V (KV) and kringle IV (KIV) [23,27]. KV is similar in apo(a) and plasminogen [23,27]. Although KIV is present only once in plasminogen, it is expressed in 10 different types in apo(a) [23,27]. Specifically, KIV contains one copy of KIV1 and KIV3–10, but variable copies of KIV2 (1 to >40 on each allele) [23,27]. KIV2 and its different repeats in apo(a) account for the high size apo(a) polymorphism and determine the size of each apo(a) isoform, its formation rate, and serum Lp(a) concentrations [26,27].

#### 3.2.3. Definition and Prevalence of High Lp(a)

Plasma Lp(a) levels vary widely between individuals and are largely determined by apo(a) size [28]. There is an inverse relationship between the number of KIV2 repeats of apo(a) and Lp(a) levels in plasma [29]. Consequently, small apo(a) isoforms are related to hyperLp(a) [29]. The presence of the *LPA* single-nucleotide polymorphisms rs3798220 and rs10455872 is also associated with hyperLp(a) [30,31]. Lp(a) levels double over the first year of life in parallel with the apo(a) gene, which is fully expressed from the first or second year of life [32,33]. Afterwards, Lp(a) levels are stable over time and seem not to be affected by diet, physical activity, or other environmental factors [3,34]. Thus, Lp(a) is enough to be measured once, unless a secondary cause is suspected or specific treatment is instituted [3,34].

Lp(a) measurement is challenging in clinical practice [35]. There is substantial variability in assays, partly due to apo(a) structure and variability in K-IV repeats [35]. Ideally, clinical assays should use an antibody for a unique non-repetitive epitope in apo(a), to recognize each Lp(a) particle once and report levels as nmol/L [35]. On the other hand, Lp(a) values measured by assays based on polyclonal antibodies cannot be reported in molar units [35]. As a compromise, they approximate their findings by comparison with apo(a) isoform-insensitive reference methods that use molar units; in case this is impossible, Lp(a) values should be reported in mg/dL [35].

Traditional thresholds for hyperLp(a) are >30 mg/dL (>75 nmol/L), with about 20% of the general population having Lp(a) >50 mg/dL (>120 nmol/L) [3,35,36].

The cholesterol contained in Lp(a) particles cannot be separated from that in LDL particles and is thus totally reported as LDL-C concentration [35]. The analyses of isolated Lp(a) particles have shown so far that cholesterol accounts for 30–45% of Lp(a) mass concentration [35]. Therefore, it has been suggested that Lp(a)-cholesterol (Lp(a)-C) can be estimated by multiplying the Lp(a) mass by 0.3, whereas corrected LDL-C is equal to measured LDL-C minus Lp(a)-C [35]. However, this approach has limitations [35]. The direct measurement of Lp(a)-C relative to Lp(a) mass has shown inter- and intraindividual variation ranging from 6% to 60%, which may affect the prediction risk [35]. Therefore, the routine correction of LDL-C for Lp(a)-C is not strongly recommended [35].

#### 3.2.4. Clinical Presentation of hyperLp(a)

The physiological role of Lp(a) is not thoroughly understood. Evidence from experimental, observational, and genetic studies has demonstrated that hyperLp(a) is an established risk factor for CHD, ischemic stroke, peripheral artery disease, heart failure, calcific aortic valve stenosis, and retinopathy in diabetic patients [3,34,35,37,38,39,40,41].

Recent guidelines recommend that Lp(a) levels should be measured at least once in each adult’s lifetime to identify those with hyperLp(a) [10]. Moreover, Lp(a) measurement should be considered in patients with personal or family history of premature ASCVD, and family history of high Lp(a) or FH [10,35]; premature atherosclerosis can be easily detected by endothelial dysfunction and early carotid lesions [42]. Screening for Lp(a) is also recommended in youth with a history of ischemic stroke and no other identifiable risk factors [35].

#### 3.2.5. Available and Upcoming Therapies for hyperLp(a)

There are neither known nonpharmacologic methods nor any medications specifically approved for lowering Lp(a) levels [23]. However, some currently used therapeutic agents have a limited effect on Lp(a) [23]. Although low-saturated fat diets and statin therapy have been previously considered to raise Lp(a) levels by approximately 10–30% [4,23,43,44,45,46] a secondary analysis of the Familial Hypercholesterolemia Expert Forum (FAME) study including a Japanese nationwide cohort of FH patients has recently shown opposing results [47]. According to this analysis, the Lp(a) levels tended to lower in those under treatment (*n* = 399) after 2–4 years of follow-up compared with the baseline values [47].

On the other hand, lipoprotein apheresis is highly effective in reducing Lp(a) levels (25–40%) [4,23]. Similarly, fibrates, niacin, lomitapide, PCSK9 and cholesteryl transfer protein (CETP) inhibitors, aspirin, antibodies to interleukin-6, nutraceuticals, tibolone, and ezetimibe moderately decrease Lp(a) levels [4,23,48].

Novel medicines based on antisense oligonucleotides (ASOs) and small interfering RNAs (siRNA) technology are currently in clinical development [4,23,49,50]. Pelacarsen, an ASO, has shown much promise with reductions of up to 92.4% in Lp(a), olpasiran, a siRNA, reduced Lp(a) with observed maximal percent reductions of >90% in a phase I study, whereas the siRNA SLN360 also reduced plasma Lp(a) concentrations in a dose-dependent way [4,23,49,50].

## 4. Discussion

### 4.1. FH and hyperLp(a)

#### 4.1.1. Prevalence of hyperLp(a) in FH Patients

Several studies have addressed whether hyperLp(a) is more common in FH patients compared with the general population (Table 1) [6,7,51]. Indeed, elevated Lp(a) levels have been observed in clinically and genetically diagnosed FH [6,7,51]. This could be attributed to decreased plasma Lp(a) clearance due to reduced LDLR function in FH [6,7,51]. Nevertheless, LDLR uptake is not the major pathway of Lp(a) clearance [52]. Indeed, statins enhance LDLR function, but increase circulating Lp(a) levels and decrease fibrinogen concentrations [53]. On the other hand, PCSK9 inhibitors, which also enhance LDLR upregulation, are associated with reductions in Lp(a) levels by 20–30% [52].

Another possible explanation could be a clinical misdiagnosis of FH due to the high Lp(a)-cholesterol content in patients with hyperLp(a) [7]. In a large cohort with 46,200 people from the Copenhagen General Population Study, ~25% of those originally considered as FH patients (*n* = 3266) did not fulfill FH criteria after adjusting LDL-C for high Lp(a) [7]. The Lp(a) concentrations between those fulfilling the clinical criteria for FH and those who did not differed significantly after adjusting LDL-C for Lp(a) levels (Table 1) [7]. Likewise, a study (*n* = 907) has shown that the proportion of FH patients diagnosed with DLCN and Simon Broome (SB) criteria decreased significantly in patients with hyperLp(a) when the LDL-C concentration was adjusted for the cholesterol content of Lp(a) (Table 1) [54]. Another large study including clinically diagnosed FH patients (*n* = 391) and individuals with genetically diagnosed FH from the general population (UK Biobank; *n* = 37,486) concluded that FH was not the cause of hyperLp(a), but it was more common for someone with hyperLp(a) to be misdiagnosed clinically with FH [8] (Table 1). The authors recalculated the DLCN scores after adjusting for the contribution of Lp(a) to LDL-C values and found that 16.6% of patients fell into a lower DLCN category [8]. In this context, the routine correction of LDL-C for Lp(a)-C is recommended in clinically suspected FH and elevated Lp(a) levels, where correction may refine or exclude diagnosis and avoid unnecessary genetic sequencing [35].

Genetically, FH and Lp(a) are independently inherited, with hyperLp(a) being much more common than FH [55]. Nevertheless, the findings derived from genetic studies are controversial regarding their relation. A large Spanish cohort study including genetically confirmed HeFH patients and non-FH individuals showed significantly higher levels of Lp(a) in the former group (Table 1) [6]. Of note, patients carrying null LDLR mutations had higher Lp(a) levels compared with those carrying defect LDLR mutations (Table 1) [6]. Another study comparing 240 genetically HeFH patients with 4015 control patients presented to a University Hospital in Japan for any reason showed that Lp(a) was significantly higher in the former group independently of the FH mutation type (Table 1) [56]. Interestingly, the Lp(a) levels did not differ between patients with PCSK9-gain of function mutations and those with LDLR ones [56]. A significant difference was also found in a study at Fuwai Hospital between 255 genetically confirmed HeFH patients and 255 healthy controls (Table 1) [57]. On the other hand, the Copenhagen General Population Study (*n* = 46,200) showed that Lp(a) concentrations were similar within the genetic heterogenicity of the FH group (Table 1) [7]. Likewise, no difference was found in the Lp(a) levels between carriers and non-carriers of FH mutations within the British Columbia cohort (*n* = 391) and the UK Biobank (*n* = 37,486, Table 1) [8].

The available evidence supports that HoFH patients have higher Lp(a) compared with non-FH hyperlipidemic individuals [58,59]. In a study analyzing 69 members of 22 families for LDLR mutations, apo(a) genotypes/isoforms and Lp(a) plasma levels, HoFH patients with two nonfunctional LDLR alleles (*n* = 26) had higher Lp(a) levels compared with HeFH ones (*n* = 43) (49.9 vs. 29.9 mg/dL) [60]. This increase was not explained by any differences in apo(a) allele frequencies, since KIV allele repeats did not differ between the two groups [60]. Similarly, in a study including 34 HoFH, 63 HeFH patients, and 22 unaffected family members, the median Lp(a) levels were higher in those with HoFH compared with HeFH and unaffected relatives (47.3 vs. 24.4 vs. 19.9 mg/dL, respectively) [61].

**Table 1:** Studies investigating prevalence of high Lp(a) levels in patients with FH.

Authors	Sample Size	Country	Diagnosis of FH	Results
Utermann et al. [62]	381	UK	Clinical	102 FH patients vs. 279 healthy subjects: 41.3 vs. 14.1 mg/dL, *p* < 0.001.
Langsted et al. [7]	46,200	Denmark	Clinical and Genetic	42,934 Unlike vs. 1675 Possible vs. 184 Probable/Definite FH patients: 23 (22.8–23.3) vs. 32 (31–34) vs. 35 (29–41) mg/dL, *p* < 0.05 (using unadjusted LDL-C for Lp(a)-cholesterol). 43,699 Unlike vs. 2360 Possible vs. 141 Probable/Definite FH patients: 24 (23.5–24.1) vs. 22 (21–24) vs. 21 (16–26) mg/dL, *p* = 0.46 (after adjusting LDL-C for Lp(a)-cholesterol). Lp(a) concentrations were similar in those with and without FH mutations: 24 (23.4–14) mg/dL in 46,124 individuals without an FH mutation vs. 23 (9–36) mg/dL in 27 individuals with an LDLR mutation (*p* = 0.10 vs. no known mutation) vs. 21 (14–28) mg/dL in 49 individuals with an apolipoprotein B mutation (*p* = 0.52) and 22 (15–28) mg/dL in 76 individuals with any FH mutation (*p* = 0.64)
Chan et al. [54]	907	N/A	Clinical and Genetic	74 patients with FH (8.2%) were reclassified to unlike FH when LDL-C was corrected for Lp(a)-cholesterol. There were no significant differences detected in the proportion of pathogenic FH mutations (27.9% vs. 33.1%) between patients with increased and normal Lp(a) concentrations at a cutoff of 50 mg/dL (*p* = 0.05).
Trinder et al. [8]	37,877	UK	Clinical and Genetic	British Columbia FH and Familial Combined Hyperlipidemia cohort; 391 FH patients vs. 245 non-FH patients: 28.7 (10.3–75.4) vs. 13 (10–48.9) mg/dL, *p* < 0.01. No significant differences were noted between carriers of a pathogenic variant in the LDLR or apolipoprotein B and noncarriers (1.43 log mg/dL vs. 1.42 log mg/dL, *p* = 0.97). UK Biobank cohort (*n* = 37,486); 221 patients with FH mutation vs. 37,265 without FH mutation: 10.7 (4.9–26.3) vs. 8.7 (4.0–25.8) mg/dL, *p* = 0.24.
Kraft et al. [60]	69	South Africa	Clinical and Genetic	26 HoFH patients vs. 43 HeFH relatives: 36.6 vs. 14.4 mg/dL, *p* = 0.004.
Li et al. [51]	8050	China	Clinical and Genetic	6250 Unlikely vs. 1519 Possible vs. 281 Probable/Definite FH patients: 51.8 vs. 57.1 vs. 60.5 mg/dL, *p* < 0.001.
Leitersdorf et al. [63]	216	N/A	Clinical and Genetic	99 FH patients vs. 117 controls: 33 vs. 22 mg/dL, *p* < 0.001.
Mbewu et al. [64]	277	UK	Clinical and Genetic	89 HeFH patients vs. 109 normocholesterolemic controls vs. 40 healthy controls: 22.7 vs. 10.0 vs. 9.1 mg/dL, *p* < 0.05.
Alonso et al. [6]	2917	Spain	Genetic	1960 HeFH patients vs. 957 non-FH relatives: 23.6 (9.6–59.2) vs. 21.0 (7–47.2) mg/dL, *p* < 0.001. 500 FH patients with null mutations vs. 246 FH patients with defect LDLR mutations: 24.4 vs. 21.5 mg/dL, *p* < 0.05.
Tada et al. [56]	4255	Japan	Genetic	198 FH patients with LDLR variants vs. 42 with PCSK9 variants vs. 4015 controls: 12.6 (9.4–33.9) vs. 21.1 (11.7–34.9) vs. 5 (2.7–8.1) mg/dL, *p* = 0.002 for the comparison between FH-LDLR or FH-PCSK9 with control group.
Sun et al. [57]	510	China	Genetic	259 HeFH patients vs. 255 matched non-FH controls: 28.9 (13.2–64.8) vs. 11.7 (5.3–26.9) mg/dL, *p* < 0.05.
Guo et al. [59]	48	France	Genetic	8 HoFH patients vs. 18 healthy subjects: 50 ± 32 vs. 20.6 ± 5.2 mg/dL, *p* < 0.001.
Sjouke et al. [61]	119	Netherlands	Genetic	22 unaffected relatives vs. 63 HeFH vs. 34 HoFH patients: 19.9 (11.1–41.5) vs. 24.4 (5.9–70.6) vs. 47.3 (14.9–111.7) mg/dL, *p* = 0.150.
Lingenhel et al. [65]	203	South Africa	Genetic	103 FH patients vs. 100 non-FH relatives: 35.4 ± 31 vs. 20.7 ± 18.1 mg/dL, *p* = 0.0014.
Wiklund et al. [66]	120	Sweden	N/A	47 HeFH patients vs. 47 controls matched for age and sex: 2.4 (2.5–124.5) vs. 9.7 (0.7–104) mg/dL), *p* < 0.001.

Abbreviations: FH: familial hypercholesterolemia; HeFH: heterozygous FH; HoFH: homozygous FH; LDL-C: low-density lipoprotein cholesterol; LDLR: low-density lipoprotein receptor; Lp(a): lipoprotein(a); N/A: not applicable; PCSK9: proprotein convertase subtilisin/kexin type 9.

The discrepancies noticed in the studies including genetically diagnosed patients with FH could be attributed to their different sample size, control groups, and settings in which they were conducted. For instance, the increased Lp(a) levels in the index cases of genetic FH referred to a lipid clinic might be ascribed to ascertainment bias, because such patients are more likely to have larger increases in LDL-C and probably also Lp(a) concentrations than do individuals with genetic FH in the general population [67]. In addition, no genetic study has found any association between Lp(a) levels and different FH variants. This rejects the theory that the impaired LDL receptor function in FH could result in the mediated catabolism of Lp(a), as already supported by kinetic studies in HoFH [67]. The analysis of the British Columbia cohort and a phenome-wide association study of Lp(a) genetic score in the UK Biobank demonstrated that hyperLp(a)-related polymorphisms are associated with a similar phenotype of FH, including increased LDL-C and a personal or parental history of premature ASCVD [8]. These results are consistent with the concept that elevated Lp(a) increases the likelihood that an individual will be diagnosed with FH. In this context, more population-based studies free of ascertainment bias with available genetic data are needed to elucidate the prevalence of hyperLp(a) in patients with FH.

#### 4.1.2. Joint Effect of FH and hyperLp(a) on Cardiovascular Risk

Studies in FH patients have demonstrated an independent association of elevated Lp(a) and CHD and stroke risk [6,51,68,69]. This association is substantially higher in individuals with previous ASCVD [70,71,72,73]. A meta-analysis of eight studies (two cross-sectional and six cohort studies) including 8378 FH subjects and reporting 1458 ASCVD outcomes demonstrated that hyperLp(a) was a significant ASCVD risk factor (relative risk: 1.97, 95% CI: 1.57–2.46) [74]. The cardiovascular risk in such patients is additionally modulated by several other genetic factors, such as the rs2048327 variant, a single nucleotide polymorphism in the SLC22A3 gene [75]. Indeed, a study including 668 HeFH patients demonstrated that the rs2048327 variant is associated with hyperLp(a) as well as with increased ASCVD risk (OR: 1.96, 95% CI: 1.21–3.19, *p* = 0.007) [75].

#### 4.1.3. Barriers to the Identification of FH

Despite the evidence-based guidelines for its diagnosis and treatment, FH remains underdiagnosed in clinical practice [2,76]. According to the global Familial Hypercholesterolemia Society (FHSC) registry, including 42,167 adults from 56 countries, the rate of FH identification was low [2]. The mean diagnosis age globally was 43 years in men and 46 years in women, with <50% of adult cases diagnosed before 40 years of age and only 2% diagnosed before the age of 18 years [2]. This might happen due to physician- or patient-related issues, such as physicians’ lack of education on FH or experience with information technology-based diagnostic algorithms, concerns about genetic discrimination, psychological consequences related to genetic diagnosis, gender, race or ethnicity, and the time/cost burden [76]. The late diagnosis of FH might also be attributed to the lack of early screening programs, considering that detection globally tends to rely on finding an index case, opportunistic screening such as health checks, or the investigation of isolated findings of an elevated LDL-C measurement [2]. Indeed, any form of cascade testing in the FHSC registry led to an earlier identification of non-index cases with fewer cardiovascular risk factors and lower ASCVD prevalence when compared with the index FH patients [2]. On the other hand, the national program of FH screening in the Netherlands mostly relying on case finding and opportunistic screening led to much earlier FH diagnosis with a lower cardiovascular burden [2]. Thus, this evidence reinforces the value of wide screening programs supported by appropriate policies and resources to identify larger numbers of patients with FH, and earlier when they might be more healthy [2]. However, such policies raise challenges about accessibility and cost, particularly in low-income and middle-income countries. In such cases, global actions are needed to promote educational campaigns, ensure a more affordable and accessible health system, and implement policies leading to the early identification of FH.

#### 4.1.4. Cascade Lp(a) Testing in FH Patients

Based on the aforementioned evidence, it has been discussed whether the incorporation of assessment of Lp(a) in FH cascade testing is feasible and should be a crucial part of models of care for FH [57,77,78]. In this context, an analysis of SAFEHEART (Spanish Familial Hypercholesterolemia Cohort Study), including 2927 family members from 755 index FH cases, investigated whether testing for Lp(a) was effective in detecting and risk-stratifying individuals participating in a FH cascade screening program [78]. Systematic screening from index cases with both FH and elevated Lp(a) identified one new case of hyperLp(a) (≥50 mg/dL) for every 2.4 screened [78]. On the other hand, opportunistic screening from index cases with FH and Lp(a) < 50 mg/dL identified one individual for every 5.8 screened, similar to that of general population [78]. Therefore, testing for hyperLp(a) during cascade FH screening seems to be effective in identifying relatives with hyperLp(a), especially in cases where the proband has both FH and hyperLp(a) [78].

## 5. Conclusions

The prevalence of hyperLp(a) is high in patients with FH. The available evidence suggests that FH and hyperLp(a) are not genetically associated, but their similar phenotype increases the likelihood of their misdiagnosis. In this context, more population-based studies with available genetic data, free of ascertainment bias, are needed to elucidate the association between FH and Lp(a). On the other hand, their combination undoubtedly multiplies the ASCVD risk, therefore the early identification of both diseases is imperative in clinical practice. Physicians’ awareness, along with cascade testing in patients with FH or hyperLp(a) or national screening programs could help identify these patients at high cardiovascular risk and tailor appropriate therapeutic strategies.

## Abbreviations

FH, familial hypercholesterolemia; LDL, low-density lipoprotein; LDLR, low-density lipoprotein receptor; ApoB, apolipoprotein B; PCSK9, proprotein convertase subtilisin kexin 9; LDLRAP1, low-density lipoprotein receptor adaptor protein 1; Lp(a), lipoprotein(a); hyperLp(a), elevated lipoprotein(a); ASCVD, atherosclerotic cardiovascular disease.
